# EAGER: efficient ancient genome reconstruction

**DOI:** 10.1186/s13059-016-0918-z

**Published:** 2016-03-31

**Authors:** Alexander Peltzer, Günter Jäger, Alexander Herbig, Alexander Seitz, Christian Kniep, Johannes Krause, Kay Nieselt

**Affiliations:** Center for Bioinformatics (ZBIT), Integrative Transcriptomics, Eberhard-Karls-Universität, Sand 14, Tübingen, 72076 Germany; Institute for Archaeological Sciences, Archaeo- & Palaeogenetics, Rümelinstraße 23, Tübingen, 72074 Germany; Senckenberg Center for Human Evolution and Palaeoenvironment, Rümelinstraße 23, Tübingen, 72074 Germany; QNIB Inc, Ortfeld 1, Böddenstedt, 29556 Germany; Max Planck Institute for the Science of the Human History, Kahlaische Straße 10, Jena, 07745 Germany

**Keywords:** aDNA, Bioinformatics, Authentication, aDNA analysis, Genome reconstruction

## Abstract

**Background:**

The automated reconstruction of genome sequences in ancient genome analysis is a multifaceted process.

**Results:**

Here we introduce EAGER, a time-efficient pipeline, which greatly simplifies the analysis of large-scale genomic data sets. EAGER provides features to preprocess, map, authenticate, and assess the quality of ancient DNA samples. Additionally, EAGER comprises tools to genotype samples to discover, filter, and analyze variants.

**Conclusions:**

EAGER encompasses both state-of-the-art tools for each step as well as new complementary tools tailored for ancient DNA data within a single integrated solution in an easily accessible format.

**Electronic supplementary material:**

The online version of this article (doi:10.1186/s13059-016-0918-z) contains supplementary material, which is available to authorized users.

## Background

In ancient DNA (aDNA) studies, often billions of sequence reads are analyzed to determine the genomic sequence of ancient organisms [1–3]. Newly developed enrichment techniques utilizing tailored baits to capture aDNA fragments, even make samples accessible that were previously both economically as well as technically unsuitable to be analyzed. The crucial step is no longer the production of genomic data from the past, but the computational reconstruction of ancient genomes using high-throughput sequencing (HTS) data, which is usually done employing short read alignment methods such as BWA [4] and standard analysis toolboxes such as SAMtools [5] or the Genome Analysis Toolkit (GATK) [6]. However, aDNA shows several characteristics, such as low endogenous DNA content, short fragment lengths, and misincorporation patterns [7], making the application of modern alignment methods with default parameters difficult. Therefore, specialized methods tailored to address the characteristics of aDNA need to be applied, to reconstruct ancient genomes successfully.

Until today, there have only been a few contributions towards a general framework for this task, such as the collection of tools and respective parameters proposed by Martin Kircher [8]. However, most of these methods have been developed for mitochondrial data in the context of the Neanderthal project [1, 9], and therefore do not scale well to larger genomes. Another contribution towards a fully automated approach has been made with PALEOMIX [10]. PALEOMIX offers separate toolkits for the analysis of aDNA samples, mapping reads, and subsequent genotyping combined with taxonomic as well as metagenomic profiling. Therefore, PALEOMIX is already a great improvement over simple scripts in a way that it provides users with access to more advanced methods and keeping these utilizable in a standardized way.

Especially for ancient bacterial research projects, this provides an improvement over former methods, which solely consist of sets of small scripts and which were therefore more prone to error and not very user-friendly. In this highly interdisciplinary field, where many users have a background in molecular biology or archaeology, the practical applicability of available methods is of high importance. The execution of scripts and their complex configurations are difficult for many researchers in this field, in particular if this requires learning programming language syntax for execution. Further barriers include the necessity to compile the source code of the underlying methods, making the installation and maintenance of sophisticated pipelines difficult even for administrative users with more bioinformatics expertise. User-friendly bioinformatic tools and methods with an interactive interface provide archaeologists and biologists with the ability to analyze large HTS data sets. For these reasons, a data-processing pipeline should be designed as comprehensively as possible, to make the underlying methods easily accessible for a wide range of users.

To address this need, we have developed EAGER, a fast and highly user-friendly next-generation sequencing (NGS) analysis pipeline for the efficient reconstruction of ancient genomes, which is designed to be used by researchers without the requirement to apply scripting languages or obtain further programming knowledge.

## Implementation

**Fig. 1:**
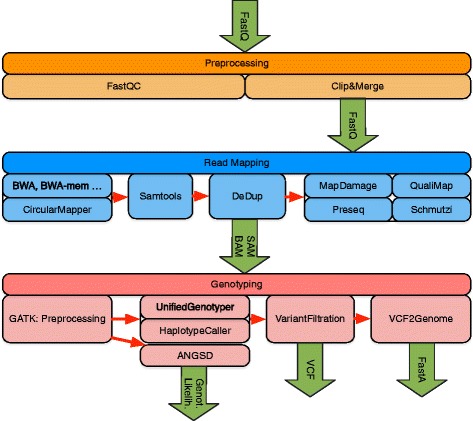
Workflow diagram of the EAGER pipeline. The pipeline consists of three distinct main components for processing and analysis of NGS data: preprocessing, read mapping, and genotyping

**Fig. 2:**
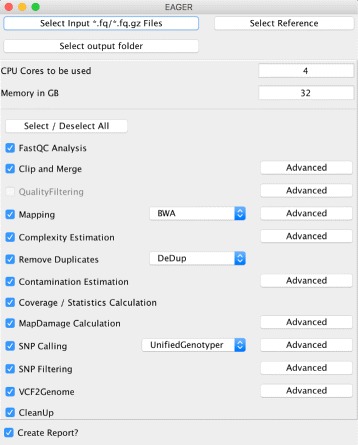
The GUI of the EAGER pipeline. The methods that can be used in the EAGER pipeline can be selected by the user and settings for each method can be adapted via the advanced buttons

EAGER consists of tools addressing read preprocessing, read mapping, PCR duplicate removal, and genotyping large-scale NGS data from NGS platforms (e.g., Illumina HiSeq, MiSeq, or NextSeq), with a specific focus on aDNA (see Fig. 1). Though the focus of EAGER lies on aDNA analysis, also DNA from any modern sample can be analyzed with it. When compared with PALEOMIX as a direct competitor, an important aspect of EAGER’s user-friendliness is that it offers a graphical user interface (GUI) that allows the user to configure the pipeline (see Fig. 2). Moreover, it integrates more tools and methods for preprocessing, analysis, and authentication of aDNA, too.

For the preprocessing, EAGER encompasses all steps necessary to process HTS raw data in FastQ format, using methods for quality assessment and prefiltering as well as newly designed methods for efficient and fast read merging and clipping. Furthermore, the pipeline provides features to map reads against a reference genome using a set of state-of-the-art mapping methods such as BWA-aln [4], Bowtie2 [11], and BWA-mem [12]. The pipeline can authenticate aDNA samples based on DNA damage patterns with mapDamage [13] and includes methods that are offered by Preseq [14] to determine the complexity of sequencing libraries. Furthermore, contamination estimation and subsequent consensus sequence generation in FastA format can be done within the pipeline using schmutzi [15]. In addition, EAGER has tools to perform genotyping [6] for mid to high coverage samples, to discover, filter, and analyze variants within a single integrated solution. For low coverage samples, the pipeline encompasses the ANGSD method to generate genotype likelihoods [16]. Furthermore, methods specifically designed for aDNA projects can be turned off, permitting the same pipeline to be used for modern DNA projects as well.

Within EAGER, we have also added four new tools, Clip&Merge, the CircularMapper, DeDup, and VCF2Genome, replacing or complementing existing tools for preprocessing, mapping, PCR duplicate removal, and genome reconstruction, respectively.

The Clip&Merge method performs a highly efficient adapter clipping of sequencing reads and subsequent merging of paired-end reads with negative insert sizes (an overlap between two sequencing reads derived from a single DNA fragment) into a single “collapsed” read.

The CircularMapper method performs an improved mapping of sequencing reads to circular reference genomes. Using the CircularMapper enables researchers to apply, for example, mitochondrial (mtDNA) haplogroup assignment methods such as HaploFind [17] with higher certainty, as many phylogenetically informative positions can be found at the beginning and the end of the mtDNA reference sequence.

**Fig. 3:**
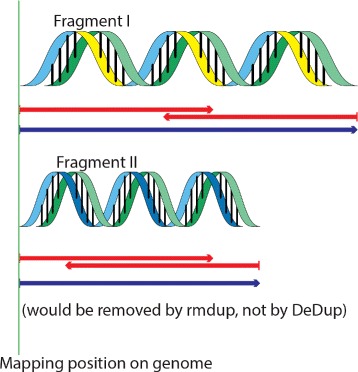
Conceptual idea of the DeDup method. Paired-end forward and reverse reads resulting from two fragments are drawn in *red* and merged reads are drawn in *blue*. Although the two merged reads stem from two different DNA fragments, SAMtools rmdup removes the read with the lower overall sum of base qualities, as only the starting position of the mapped reads is taken into account. DeDup takes both mapping positions (start and end) into account, and in this case would keep both reads

Another contribution is the DeDup method, which removes duplicates and is tailored specifically to merged paired-end reads. DeDup considers specific properties of merged reads that are not considered by already existing methods, such as rmdup in SAMtools [5], by taking both ends of the fragment into account (see Fig. 3).

Finally, we incorporated our new VCF2Genome tool into the EAGER pipeline, which can take variant calls from the genotyping step and generate a draft genome sequence, with specific filtering criteria applied to each call performed by the genotyping method. The generated draft sequence can then be used by other methods, e.g., for performing phylogenetic analyses.

The pipeline also has a method that automatically produces a comprehensive report of the processed data, making the retrieval of statistics about generated data as easy as possible.

EAGER has been implemented in the Java programming language and can be run on several types of operating system, including but not limited to desktop workstations. Setting up the pipeline has been realized using Linux containers via Docker [18], to provide users and administrators with a portable and flexible distribution of the pipeline, without complex configuration scripts or the need to compile the source code themselves. Once set up, the pipeline can be accessed via a GUI (see Fig. 2). The GUI is applied to configure the analysis tasks, hiding most of the complexity from the user. For advanced users, options for more detailed parameter adjustments exist. Previously published protocols, such as PALEOMIX [10], partially overlap in terms of features; however, EAGER offers an improved user experience by providing a GUI, swift setup, and short processing time.

## Results and discussion

**Fig. 4:**
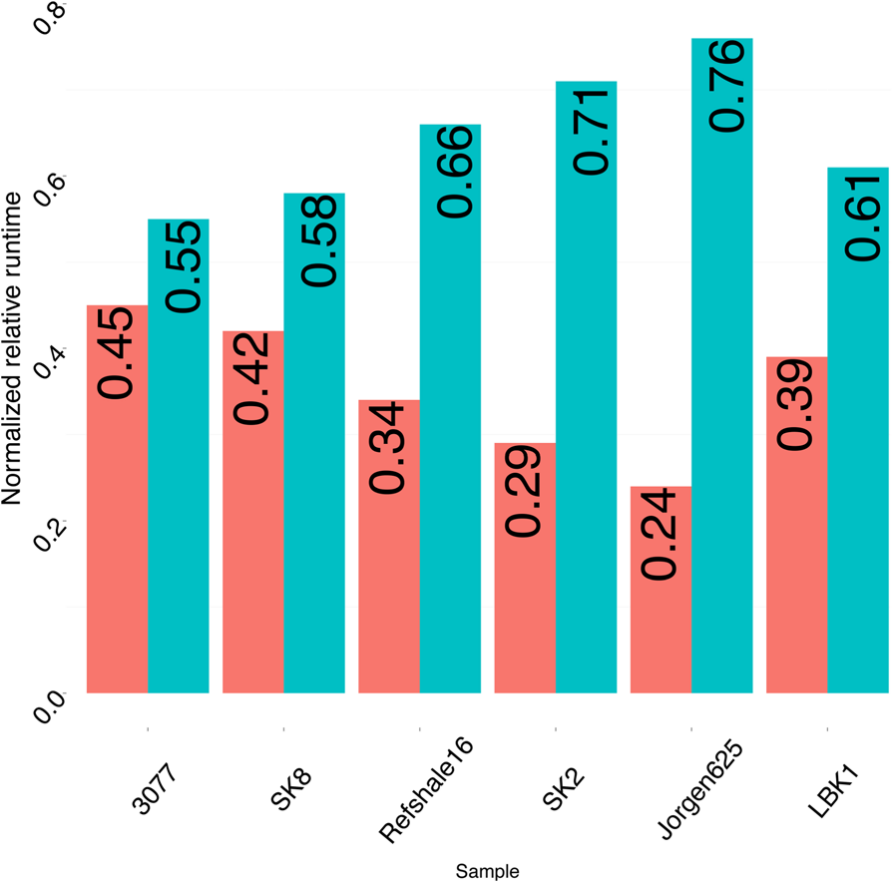
Run-time comparison of EAGER and PALEOMIX. Normalized run times are shown for six data sets: five ancient leprosy data sets [2] and an ancient human sample [19]. EAGER (*red*) performs on average 1.53 times faster than the PALEOMIX (*turquoise*) pipeline (see Table 2 for the absolute run times and respective factors of each sample)

EAGER has been implemented such that processes are executed in parallel whenever the underlying methods support this and it is optimized to store the generated output in compressed file formats, making the pipeline both CPU and storage efficient. To evaluate the performance of the pipeline and the fundamental tools, EAGER has been applied to six published data sets: five ancient *Mycobacterium leprae* data sets from Schuenemann et al. [2] and a high coverage ancient human genome data set published in Lazaridis et al. [19] (see Table 1). We compared EAGER to PALEOMIX, currently the most comprehensive protocol for aDNA, which provides two distinct and independent pipelines: a mapping pipeline and a phylogenetic pipeline to generate BAM files and perform genotyping together with downstream phylogenetic analysis. EAGER features more tools and methods than PALEOMIX, including initial raw sequencing quality assessment with FastQC, library complexity estimation with Preseq, and several new methods such as Clip & Merge, CircularMapper, and DeDup combined with QualiMap for mapping statistics. The mapping pipeline and parts of the phylogenetic pipeline of PALEOMIX have been applied to the test data sets to assess the run-time performance in comparison to EAGER. Some of these features have been turned off, as for example Preseq, as these differ too much for direct comparison with PALEOMIX. EAGER and PALEOMIX have been executed with default parameters where applicable, setting mapping parameters to the same values to ensure comparability. EAGER runs on average 1.53 times faster than PALEOMIX on the evaluated data sets (see Fig. 4 and Table 2). As both PALEOMIX and EAGER use similar mapping methods (e.g., BWA), this is mainly due to our new and improved read trimming, merging, and de-duplication algorithms.

**Table 1:** Sample names, corresponding SRA/ENA identifiers, number of reads, read length, number of bases, and the average fragment length for the samples used for evaluation of the EAGER pipeline

Sample	SRA ID/ENA ID	# of reads	Read length	# of bases	Avg. fragment length
3077	SRX275526	6,029,646	76	916,506,192	60.87
Refshale16	SRX276068	39,915,365	76	6,067,135,480	79.91
Jorgen625	SRX275549	15,101,591	200	6,040,636,400	164.24
SK2	SRX275535	54,243,849	100	10,848,769,800	62.84
SK8	SRX275538	9,898,159	76	1,504,520,168	81.41
LBK1	SAMEA2697125	227,266,922	101	45,907,918,244	69.71
LBK2	SAMEA2697125	222,751,961	101	44,995,896,122	69.69
LBK3	SAMEA2697125	227,779,612	101	46,011,481,624	69.72
LBK4	SAMEA2697125	207,406,901	101	41,896,194,002	69.72
LBK5	SAMEA2697125	207,983,311	101	42,012,628,822	69.67
LBK6	SAMEA2697125	208,835,520	101	42,184,775,040	69.71
LBK7	SAMEA2697125	213,784,583	101	43,184,485,766	69.68
LBK8	SAMEA2697125	228,184,096	101	46,093,187,392	69.71

For the LBK data set, we chose to evaluate a single lane of data (LBK1), as the other samples (LBK2–8) showed very similar features

**Table 2:** Execution times (in seconds) of the EAGER and PALEOMIX pipeline applied to five ancient *Mycobacterium* leprosy data sets and eight *Homo sapiens* data sets (LBK1–LBK8) (see Table 1)

Data set	EAGER	PALEOMIX	Factor
LBK1	57,853	90,181	1.55
LBK2	61,066	88,526	1.44
LBK3	58,252	90,032	1.54
LBK4	54,215	82,318	1.51
LBK5	53,676	82,500	1.53
LBK6	54,790	82,090	1.49
LBK7	61,859	83,544	1.35
LBK8	57,782	91,015	1.57
3077	1,066	1,310	1.22
Jorgen625	4,224	13,160	3.11
Refshale16	4,913	9,329	1.89
SK2	5,342	13,196	2.47
SK8	1,508	2,089	1.38
Average	36,657	56,099	1.53

The respective run times have been calculated using the Unix time command, stated are the real times. Execution was performed on the same host system. The parameters of both pipelines have been chosen to be as close to each other as possible, e.g., the mapping parameters have been set to the same values, where this was possible. The factor in the last column refers to the ratio of the PALEOMIX run time versus that of EAGER

**Fig. 5:**
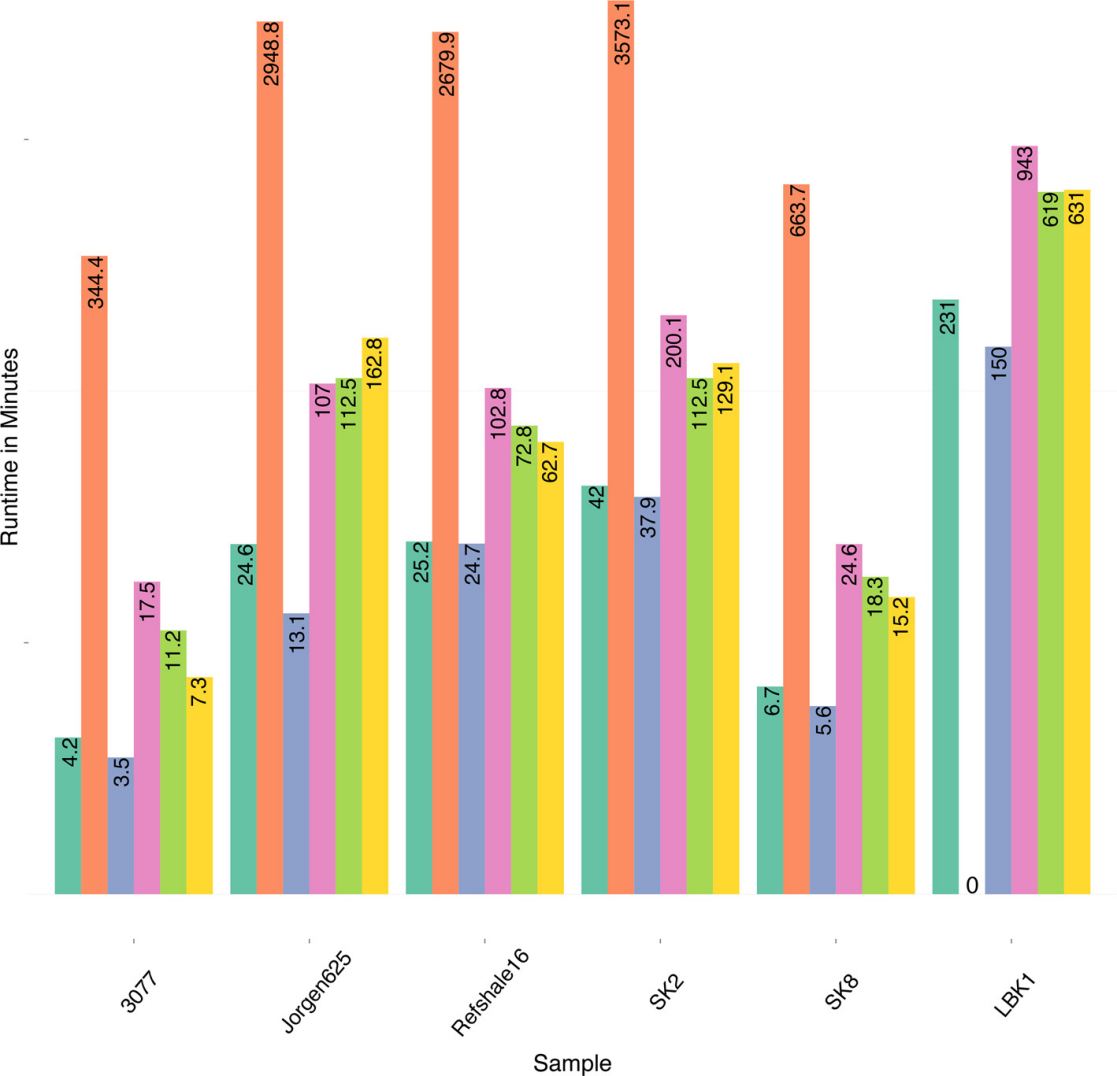
Run-time comparison of several read merging tools. Our own method Clip&Merge (*green*) was compared to MergeTrimReads (*red*), CutAdapt + FLASH (*blue*), SeqPrep (*purple*), LeeHom (*light green*), and AdapterRemoval (*yellow*). The evaluation was performed on five *Mycobacterium leprae* data sets and one exemplary human data set (LBK1). Clip&Merge outperforms the other available methods in terms of speed, except for the combination of CutAdapt and FLASH. MergeReadsFastQ was not evaluated on the LBK1 data set, due to the run-time limitations posed by the method, which is shown as a run time of zero for this case

We then evaluated our newly developed method Clip & Merge, for efficient adapter clipping and paired-end read merging in much more detail, by comparing it to six other similar and commonly used tools. For the comparison, we used the same data sets as above. Clip&Merge performs very well in terms of run time on the tested samples (see Fig. 5), furthermore providing increased mapping rates when compared to competitor tools (see Table 3). The latter is an important feature as the improved merging of aDNA reads and subsequent improved read mapping rates greatly influence further downstream analyses such as genotyping. In addition, we also evaluated the Clip & Merge application with respect to error tolerance on an artificial data set, provided by the authors of FLASH [20] for different levels of errors ranging from 0 to 5 %. The accuracy of Clip&Merge exceeds or is similar to that of its competitor tools on these simulated data sets, as can be seen in Table 4. As LeeHom uses a stochastic approach to perform adapter clipping and read merging within one step, we excluded the method from the simulation evaluation, as it only produced very low merging rates, which are most likely because the simulated data did not contain any adapter sequences and LeeHom was not able to perform on such data sets without adapters. Not all the methods have been evaluated on all data sets, as, for example, MergeReadsFastQ is substantially slower than other methods that forbid the application on a human genome data set like the one from Lazaridis et al. [19].

**Table 3:** Mapping rates for different adapter clipping and read merging methods

Sample	Method	Number of	Mapped
		mapped reads	reads [%]
3077	Clip&Merge	1,043,672	17.31
	MergeTrimReads	1,006,194	16.7
	CutAdapt + FLASH	1,036,940	17.2
	SeqPrep	949,073	15.74
	LeeHom	981,558	16.28
	AdapterRemoval	931,529	15.45
Jorgen625	Clip&Merge	2,703,428	17.9
	MergeTrimReads	2,623,243	17.37
	CutAdapt + FLASH	2,599,158	17.21
	SeqPrep	2,595,366	17.19
	LeeHom	2,617,909	17.34
	AdapterRemoval	2,524,087	16.71
Refshale16	Clip&Merge	13,368,593	33.49
	MergeTrimReads	13,812,705	34.6
	CutAdapt + FLASH	11,533,714	28.9
	SeqPrep	11,516,472	28.85
	LeeHom	8,916,759	22.34
	AdapterRemoval	11,431,717	28.64
SK2	Clip&Merge	48,536,318	89.48
	MergeTrimReads	48,610,983	89.62
	CutAdapt + FLASH	48,402,551	89.23
	SeqPrep	48,240,750	88.93
	LeeHom	48,337,919	89.11
	AdapterRemoval	47,095,207	86.82
SK8	Clip&Merge	1,283,126	12.96
	MergeTrimReads	1,280,119	12.93
	CutAdapt + FLASH	1,109,626	11.21
	SeqPrep	1,107,013	11.18
	LeeHom	908,549	9.18
	AdapterRemoval	1,100,326	11.12
LBK1	Clip&Merge	113,843,504	50.1
	CutAdapt + FLASH	52,681,090	26.8
	SeqPrep	109,491,426	51.6
	LeeHom	111,943,019	50.9
	AdapterRemoval	107,484,735	47.2

Version 1.6 of Clip&Merge was tested. Version 1.7.1 of CutAdapt was evaluated together with version 1.2.11 of FLASH. We used SeqPrep version 1.1, and MergeTrimReads and LeeHom in the versions publicly available on 10 January 2015. Version 1.5.4 of AdapterRemoval was used. For the LBK1 sample, the MergeTrimReads method was not evaluated, as the run time of the method had exceeded those of all other methods when tested on smaller data sets by far. Overall, the tools Clip&Merge and MergeTrimReads performed best

**Table 4:** Merging accuracy on simulated test data sets with ranging error rates from 0–5 %

	Accuracy				
	0 %	1 %	2 %	3 %	5 %
Clip&Merge	99.96	67.30	40.70	32.69	30.03
FLASH	97.68	66.08	40.30	32.59	30.04
AdapterRemoval	98.13	66.54	40.39	32.57	30.02
SeqPrep	97.68	44.22	33.07	30.82	30.01

The data sets were downloaded from Magoc et al. [20]

**Fig. 6:**
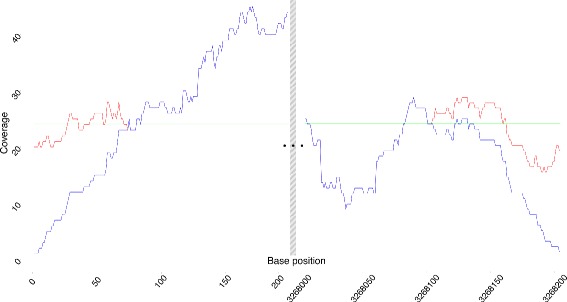
Comparison of coverage of CircularMapper and BWA. The plot illustrates the coverage of the CircularMapper method (*red*) in comparison with the coverage obtained using only the BWA method (*blue*) to reconstruct the SK8 *Mycobacterium leprae* sample. The coverages have been log2 transformed. The average coverage over the whole genome is shown in *green*. The first 200 (*left*) and the last 200 bases (*right*) of the genome are shown here to demonstrate the effect of the CircularMapper method. Because of the specific fragment length within the sample, the effect is restricted to the first and last approximately 80 bases

A further method has been implemented for circular genomes, where typically used mapping methods, such as BWA or Bowtie2, are unable to obtain even coverages at the ends of the circular reference genome due to technical limitations. Most of the mapping algorithms as of today only achieve even coverages on the interior parts of reference genomes, whereas on circular genomes they are unable to achieve even coverages at both ends of the respective reference genome. For circular genomes, the new method CircularMapper can even the coverage obtained at the ends of the circular reference genome. In the current version, CircularMapper can be used only after mapping with BWA. To demonstrate how the method evens the coverage, we have applied BWA with and without CircularMapper to one of the ancient *M. leprae* samples (Sample SK8, see Table 1). Visual inspection of the overall coverage revealed that the results obtained showed similar coverages across the reference genome, however with much more uniform distribution of the coverage at both ends of the circular reference genome when applying the CircularMapper method in addition (see Fig. 6).

**Fig. 7:**
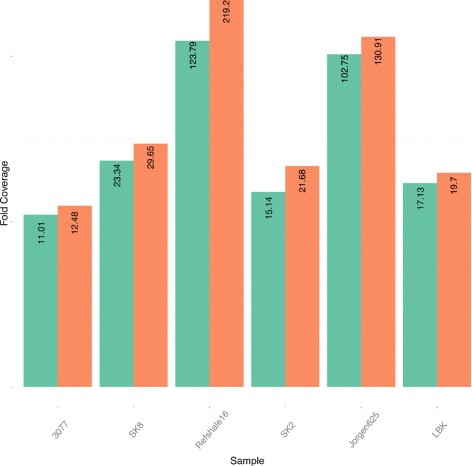
Comparison of duplicate removal methods. Coverages obtained when applying the SAMtools rmdup method (*green*) and the DeDup method (*red*) to five ancient leprosy samples and one ancient human sample

The performance of DeDup in comparison to SAMtools rmdup applied to the five ancient leprosy samples and one ancient human sample is shown in Fig. 7 and Table 5. DeDup removes duplicates on merged paired-end data with a more sophisticated approach than previous methods such as SAMtools rmdup. The improved DeDup method increases the coverage on paired-end sequencing data with negative insert sizes significantly when merging was applied. Subsequently, it improves downstream results such as variant detection and is almost as fast as rmdup from SAMtools. In addition, we performed a sub-sampling experiment on one of the data sets (Jorgen625), and then compared the performance of rmdup and DeDup both with respect to achieved genome coverages as well as single-nucleotide polymorphism (SNP) calling on low coverage data. The results (see Table 6) indicate that DeDup retains more positions than rmdup. Furthermore, the difference between using no duplicate removal at all and DeDup is small. Especially on low coverage samples (below 5–10 ×), which is rather typical for aDNA samples, the DeDup method keeps more positions than the rmdup method. This leads to a higher sensitivity of the downstream variant calling pipeline with more resolved positions, while rmdup would remove too many reads, which therefore, leads to a loss of many positions. Furthermore, on high coverage samples, DeDup achieves higher maximum coverages, which is particularly relevant for short fragment lengths, which also are typical characteristics of aDNA samples [7]. For a sample with only few variants, as is the case for the Jorgen625 sample, the differences between the different duplicate removal methods are only subtle, but for other samples with higher numbers of mutations, we expect the differences to be significantly larger.

**Table 5:** Run-time evaluation and performance evaluation of DeDup compared to SAMtools rmdup on five leprosy and eight human data sets

	Run time in seconds		Total # of mapped reads	Removed reads	
Sample	DeDup	Rmdup		DeDup	Rmdup
3077	53	42	5,737,174	410,045	492,798
SK2	146	66	51,741,310	47,353,377	47,567,518
SK8	124	109	10,597,941	189,328	323,524
Jorgen625	347	335	16,093,580	96,393	699,310
Refshale16	479	371	43,528,407	3,802,345	8,055,585
LBK1	2845	2446	227,603,728	3,138,204	6,708,814
LBK2	2779	2544	230,911,458	2,828,977	6,060,762
LBK3	2809	2440	228,167,295	3,264,858	6,719,244
LBK4	2451	2235	207,956,568	2,762,565	5,929,959
LBK5	2531	2248	208,277,143	2,802,601	5,983,802
LBK6	2572	2255	209,806,794	2,789,629	6,005,481
LBK7	2859	2532	229,979,501	2,466,574	5,293,065
LBK8	2804	2441	228,466,493	3,177,587	6,786,388

**Table 6:** Comparison of DeDup with the SAMtools rmdup method

Percentage	Method	Var calls	cov(fold)	cov(%)	refCall/ *Δ*
1	NoRMDup	1	1.16	1.02	33,277
1	DeDup	1	1.16	1.01	−207
1	rmdup	1	1.16	0.98	−1,362
2	NoRMDup	11	2.33	10.17	332,395
2	DeDup	11	2.33	10.14	−1,051
2	rmdup	11	2.32	9.85	−10,563
4	NoRMDup	55	4.7	49.82	1,628,172
4	DeDup	55	4.69	49.73	−2,978
4	rmdup	55	4.64	49.10	−23,481
5	NoRMDup	80	5.89	66.85	2,184,874
5	DeDup	80	5.88	66.77	−2,889
5	rmdup	78	5.8	66.19	−21,761
6	NoRMDup	91	7.06	78.85	2,576,795
6	DeDup	91	7.05	78.78	−2,219
6	rmdup	89	6.94	78.31	−17,500
7	NoRMDup	102	8.26	86.68	2,832,796
7	DeDup	102	8.24	86.62	−1,931
7	rmdup	101	8.09	86.29	−12,650
70	NoRMDup	114	82.58	98.39	3,215,440
70	DeDup	114	80.84	98.39	0
70	rmdup	114	68.87	98.39	−52
80	NoRMDup	114	94.38	98.4	3,215,840
80	DeDup	114	92.11	98.4	−2
80	rmdup	114	76.89	98.4	−54
90	NoRMDup	114	106.23	98.42	3,216,400
90	DeDup	114	103.36	98.42	0
90	rmdup	114	84.62	98.42	−30
100	NoRMDup	114	118.03	98.43	3,216,748
100	DeDup	114	114.51	98.43	−1
100	rmdup	114	92.02	98.43	−30

The first column describes the percentage of randomly drawn reads from the Jorgen625 leprosy data set, with a genome size of 3,268,202 base pairs. *Var calls* shows the number of variant positions that were called. *cov(fold)* and *cov(%)* show the coverage of the genome. *refCall* describes the number of reference calls that were made, where *Δ* describes the difference between the non-de-duplicated sample at the given sub-sampling degree and the duplicate removed sample. All other positions of the genome have been filtered out. The parameters to call a position confidently were a coverage of at least fivefold, a variant quality of at least 30, and a minimum allele frequency of 90 %. NoRMDup refers to not applying any duplicate removal to the corresponding sample

As the sequencing of aDNA often results in low coverage genomes, we used ANGSD-tools, as integrated in EAGER, and analyzed its performance. For this we simulated low coverage data with the full LBK/Stuttgart sample from Lazaridis et al. [19]. Using SAMtools, we randomly extracted reads from the given sample with coverages ranging from 0.09X to 7.51X. Then, we used ANGSD to reconstruct the genomes from the low coverage sub-sampled data sets. We then compared these results to the full coverage genotyping results obtained on the published ≈19X data set, as shown in Table 7. EAGER performed well in these tests and was able to reconstruct high numbers of variants when compared to the genotyping performed on the full coverage data set, showing that it could even work well on low coverage input data.

**Table 7:** Downsampling experiment for simulation of low coverage data

Sampling	0.005	0.01	0.02	0.05	0.10	0.20	0.30	0.40
Expected mean coverage	0.10	0.19	0.39	0.97	1.94	3.88	5.82	7.76
Achieved mean coverage	0.09	0.19	0.38	0.94	1.88	3.75	5.63	7.51
Cov % of genome ≥1 read	8.70 %	16.47 %	29.71 %	56.28 %	76.85 %	88.41 %	90.69 %	91.28 %
Cov % of genome by ANGSD	8.64 %	16.37 %	29.55 %	56.06 %	76.68 %	88.35 %	90.67 %	91.27 %
% of correctly called bases	99.72 %	99.73 %	99.74 %	99.77 %	99.82 %	99.89 %	99.92 %	99.93 %

The first row refers to the percentage of reads that were randomly sampled from the original data set (LBK/Stuttgart) from Lazaridis et al. [19] (coverage ≈19×) using SAMtools. The expected mean coverage was derived by multiplication of the original coverage with the sampling value. The achieved mean coverage was calculated using QualiMap after mapping. *Cov % of genome ≥1 read* is the percentage of the genome that was covered by at least one read. This was also calculated using QualiMap. *Cov % of genome by ANGSD* is the percentage of the genome that was reconstructed from the genotypes as derived with ANGSD. *% of correctly called bases* is the percentage of bases that ANGSD called correctly not regarding the base “N”

To elucidate the performance of the full EAGER pipeline, a comparison with already published results obtained on an ancient human individual from Lazaridis et al. [19] has been performed (see last row of Table 1). Already processed results were downloaded and compared to the respective results obtained when processing the raw sequencing data using the EAGER pipeline. Here, we focused on comparing the results of the genotyping analyses, more precisely the variants called by EAGER compared to those published by Lazaridis et al. Note that comparing different variant calling methods is difficult in aDNA projects, as there are no gold standards for aDNA data sets, unlike the Genome in a Bottle (GIAB) data sets for modern DNA for example [21]. This restricts the comparison to qualitative measures, such as the transition to transversion ratio, the total number of called variants, and the percentage of variants found in dbSNP [22]. EAGER performs well in terms of the computed transition to transversion (Ti/Tv) ratio (see Table 8), achieving a Ti/Tv ratio of 2.21 on the LBK1 (Linearbandkeramik) data set, whereas the published data showed a respective Ti/Tv ratio of 2.4. For variants restricted to those published in dbSNP, the Ti/Tv ratio dropped to 2.1 for both EAGER and the published data, which is exactly the expected value for human samples [23]. Additionally, 88.5 % of found variants could be verified as already published variants in dbSNP, a higher percentage than the previously published 78.8 %. The differences between the published data and the results obtained with EAGER are most likely due to updated methods within EAGER, for example, as GATK has been updated frequently in the meantime.

**Table 8:** Evaluation of the EAGER pipeline in comparison with already published data (ENA SAMEA2697125)

	EAGER	Published
Ti/Tv ratio	2.21	2.4
Ti/Tv dbSNP	2.1	2.13
Total variants predicted	4,098,642	4,340,699
Variants annotated in dbSNP	3,626,496	3,419,360
% dbSNP	88.48	78.77

For whole human genomes, a good Ti/Tv ratio is typically considered to be around approximately 2.1 and a high percentage of >80 *%* of the total found variants in a sample is expected to be found in the dbSNP database (see last row)

We compared the EAGER pipeline and PALEOMIX on a modern data set from the *GenomeComparison*&*Analytic Testing* (GCAT) platform (available from bioplanet.combioplanet.com). We used the 30X exome sequencing data set derived from the GIAB initiative to evaluate the mapping and subsequent variant calling of EAGER and PALEOMIX. The results are summarized in Table 9. It can be seen that the result produced by EAGER outperforms both the pipeline offered through GCAT as well as PALEOMIX on the level of sensitivity of the called variants at almost the same perfect level of specificity.

**Table 9:** Comparison of EAGER to a benchmark data set from GIAB

Pipeline	GIAB Sensitivity	GIAB Specificity
GCAT	85.21 %	99.9975 %
EAGER	88.21 %	99.9963 %
PALEOMIX	82.83 %	99.9962 %

*GCAT* is the result of GCAT using BWA followed by the GATK Unified Genotyper. *EAGER* is the result that was produced using EAGER and also BWA with standard parameters for the Unified Genotyper of GATK. PALEOMIX is the result that was produced using PALEOMIX using BWA and the SAMtools mpileup method to get genotypes

In summary, we have developed EAGER, a user-friendly and integrated pipeline for the efficient reconstruction of ancient genomes, providing users with easy access to a large number of state-of-the-art and complementary methods. EAGER is an actively developed pipeline that has been designed as a modular framework. Therefore, while keeping the usability aspect as its first and foremost priority, it allows for the easy integration of extended features and new tools that will contribute to high-throughput DNA sequencing data analysis in the future.

## Methods

### Preprocessing

EAGER can perform several raw read preprocessing steps, including the initial analysis of raw sequencing reads using FastQC (Andrews, S.; FastQC: A quality control tool for high throughput sequence data, unpublished, 2010) to assess the basic quality of the generated NGS data. aDNA usually suffers from post-mortem damage, with decreasing read lengths and increasing misincorporation patterns, rendering the analysis of aDNA data difficult with the currently applied NGS methods. Furthermore, the fragments are typically of smaller length than in modern data sets, making the reconstruction of a full genome even more difficult. Read merging is therefore a necessary step to improve the overall quality of reads from aDNA. Furthermore, some mapping algorithms, for example BWA-aln, have difficulties in mapping paired-end data with negative insert sizes.

The newly developed Clip&Merge method is capable of clipping adapter sequences, merging clipped paired-end reads if possible, and trimming non-merged reads based on a user-defined quality threshold. To achieve this, a clipping strategy that was motivated by the technique implemented in the FASTX-Toolkit (Gordon A, Hannon GJ: Fastx-toolkit. FastQ/A short-reads pre-processing tools, unpublished) was developed, making use of multi-core systems by running the clipping on forward and reverse reads in parallel. To identify adapter sequences at the ends of the reads, a local alignment based on the Smith–Waterman algorithm [24] between the adapter sequence and the read is calculated. All bases between the start position of the alignment and the end of the read are then removed, if the alignment fulfills the requirements defined by the user, including an allowed number of mismatches and a minimum length of the overlap region. If the start position of the alignment and the adapter are different, the start position is moved towards the 5^′^ end of the read by the number of unaligned bases at the start of the adapter sequence. This ensures that there are no adapter bases left in the read sequence, avoiding the merging of adapter sequences in the subsequent step. The merging step calculates the reverse complement of the reverse read and then performs a maximal overlap search between the ends of the forward and the reverse complemented reverse reads, starting with a maximal overlap and a pairwise comparison of the nucleotides in the overlap region. If the edit distance in the overlap region is lower than a defined threshold and the size of the overlap region is larger than a defined minimal overlap size, the merging is accepted. Bases with very low sequencing quality are treated as undefined nucleotides and do not contribute to the edit distance in the temporary overlap region. If the criteria for an overlap cannot be fulfilled properly, the temporary overlap is shifted by one base and the calculations are repeated, until either a satisfying overlap has been found or no overlap could be identified.

### Mapping

EAGER features several mapping algorithms that can be accessed and configured easily via the integrated GUI. Currently, BWA [4], BWA-mem [12], Bowtie [25], and Stampy [26] are available. Many available mapping methods are optimized towards mapping NGS reads to a linear reference genome. However, the majority of bacterial genomes as well as the human mitochondrion are circular. Methods like BWA try to map sequencing reads completely against the reference genomes and mark reads that cannot be mapped completely as unmapped. Even improved methods that allow for soft-clipping, for example BWA-mem, have not solved these issues completely. Although this does not pose an issue for reads falling into the interior regions of a circular genome, the first as well as the last couple of hundred bases of circular genomes are usually reconstructed poorly due to the inability to map reads to the respective regions. The resulting coverage in such regions has been observed to be significantly lower than the average coverage on the whole genome of the respective organism, which poses difficulties for some downstream analysis tasks such as haplotyping or full-genome reconstruction, where an even coverage of the whole genome is required [17]. To overcome these issues, the CircularMapper method has been developed.

CircularMapper performs two independent steps: It first creates an elongated reference genome, by adding the first *k* bases of the reference genome to the end of the genome and then mapping the sequencing reads against that elongated reference genome. Typically, *k* is chosen to be a default of 500 bases but can be set by the user. The elongation value *k* should be chosen to be at least the maximal read length observed in the preprocessed sequencing data set used as input. After this, reads are categorized by the second CircularMapper component into three different categories. The first category of reads is found in the region ranging from *k* to the unmodified genome length and reads in the category do not require any changes. The second category of reads is found in the first or in the last *k* bases of the modified genome. These are remapped in a separate step. This is important, as reads that are found to fit two or more regions on a reference genome are usually marked ambiguous by the mapping algorithm. As they clearly result from the modifications introduced by the CircularMapper, these reads can be remapped safely against an unmodified reference and then taken into the final mapping results. Lastly, reads that have a starting position within the unmodified reference genome and simultaneously have an end position in the modified region are considered as overlapping reads, spanning the circular overlap region of the reference. These reads are split according to their overlap and are afterward placed at their correct positions by the second component of the CircularMapper method. For human genomes, where the mitochondrion is the only part of the genome to be organized as a circular chromosome, the method can perform this extension and split approach on the whole genome, but only modifying the mitochondrion reference in such a case. This is required, because the human genome has nuclear mitochondrion DNA (NUMTs) regions [27]. Mapping DNA against only the mitochondrion reference genome would therefore result in an overestimation of actual coverage, as reads that would otherwise map to NUMTs, are mapped against the mitochondrion reference in such cases. Thus, a mitochondrion DNA reconstruction should always be performed on the full human genome to take these NUMTs into account.

To ensure that the resulting SAM and/or BAM files of aDNA sequences are processed appropriately, we developed an improved duplicate removal method called DeDup, which is integrated in the pipeline, too. As aDNA samples often show very low amounts of endogenous DNA, enrichment and amplification methods are often used to increase the number of DNA reads retrieved from the given DNA fragments [2, 3]. Unfortunately, these methods increase the number of sequencing duplicates stemming from the same fragments. Since the coverage of specific genomic loci is important for downstream analysis, the statistics of the respective loci, such as duplicates, can convey a false-positive trust in a specific region that might only result from a high number of duplicate entries. This is undesired, and therefore in silico methods are utilized to remove duplicated sequencing reads. Several methods to achieve this have been proposed, with the most prominently used being rmdup in SAMtools [5]. This method works well on regular paired-end sequencing data, where the 3^′^ end of the forward reads and 5^′^ end of the reverse reads are known. Since rmdup only considers the 5^′^ positions of the respective reads, the assumption regarding equal 3^′^ ends fails for merged paired-end reads, where the 3^′^ end is not known in advance. Thus, the method may also remove reads that stem from different fragments. To compensate for this, the DeDup method has been implemented following a principle described by Green et al. [9], which considers both the 5^′^ and the 3^′^ positions of the respective reads and thus, keeps merged reads that have different lengths (see Fig. 3). When two reads are mapped to the same start and end positions, the read with the higher sum of base qualities is kept, whereas the read with the inferior sum of base qualities is discarded accordingly. For unmerged reads, the method performs the same duplicate removal procedure as the SAMtools rmdup method for single-end reads. DeDup has been optimized to work correctly on single-end data as well as (partially) merged paired-end data with negative insert sizes or collapsed reads. For paired-end data with positive insert sizes, as for typical modern data, the EAGER pipeline features the MarkDuplicates method from the Picard toolkit to enable paired-end de-duplication for non-merged data, too. Finally, the method QualiMap [28], which reviews the overall mapping results, has been made accessible in the pipeline.

An important step during aDNA analysis is authentication. This can be addressed by damage pattern analysis and fragment length calculation. In EAGER, we have, therefore, integrated mapDamage [13] for an automated damage pattern analysis to authenticate ancient samples. Furthermore, the mapping module contains the Preseq tool [14] to determine the complexity of the sequencing library. To enable researchers to perform contamination estimation on aDNA data, which is a crucial step for assessing whether data has been contaminated with DNA from foreign sources, we also integrated the recently published method schmutzi [15] into the EAGER pipeline. Schmutzi estimates contamination based on a maximum likelihood approach using deamination patterns and fragment lengths typical for aDNA. In addition, schmutzi can be used to compute an improved endogenous human mitochondrial genome sequence by taking the estimated contamination into account.

### Genotyping

The pipeline can be used to perform a full genotyping of a given sample using GATK [6], including both available genotypers (the UnifiedGenotyper and the HaplotypeCaller) in GATK along with the GATK variant filtration method to perform downstream analysis of called variants inside the pipeline. Within EAGER, the GATK Best Practice’s Guidelines are followed [29], including IndelRealignment but excluding the Base Score Recalibration procedures. As Base Score Recalibration requires some reference VCF file to perform the recalibration properly, which rarely exists for the application on ancient genomes and applications that involve species other than humans, we excluded the method, as it could hinder the detection of potentially ancient variants that are not present in modern populations when used in aDNA projects. Furthermore, since modern sequencing machines produce very reliable base quality scores, we decided to remove the Base Score Recalibration step from the EAGER pipeline.

Furthermore, we developed the VCF2Genome method, which reads a VCF file produced by the genotyping method of choice. For each call, it incorporates one nucleotide into a new draft genome sequence. By default, if the genotyper calls a reference base and the quality of the respective call was at least 30 with a minimal coverage of the respective position of at least five reads, then a reference base is included in the draft genome sequence at this position. If a variant was called (SNP), it is included if the same quality threshold is fulfilled, at least five reads covering the respective locus contain the respective SNP, and the fraction of mapped reads containing the SNP was at least 90 %. If not all of these requirements are fulfilled, but the quality threshold is still reached, the reference base is called instead, but only if it is confirmed by at least five reads and contained in 90 % of the reads covering the locus. The stated thresholds and filtering criteria are the current default values set by the pipeline and can be configured by the user. If neither the reference call nor a variant call can be made, the character “N” is incorporated at the position. To keep the potential introduction of too many “N” characters due to sequencing errors as low as possible for low coverage genomes, the major allele is still regarded as being confirmed by 100 % of the reads, if there is only a single read confirming the minor allele. Additionally, the tool produces two further draft sequences. The first contains the reference base instead of “N” in all cases, whereas the second contains a special uncertainty encoding. Instead of the “N” character, it contains lower-case letters “a”, “c”, “g” and “t” at positions where a call was rejected, for example due to low coverage but the reads covering the respective position unambiguously indicate a SNP call. For uncertain reference calls, an “R” is inserted. Using this approach, users can differentiate between a clear SNP call, a weak SNP call, a clear/weak reference call, and no call at a certain position more effectively. As many samples in aDNA projects only show low coverages, EAGER also features the ANGSD method [16] to create genotype-likelihood-based output on low coverage data, using an already established method.

### Report generation

Additionally, EAGER features a report engine that can be used to generate summary reports with the most important statistics including mapping and genotyping of all processed samples (see Table 10 for an excerpt and Additional file 1 for the full table). This offers the possibility of assessing the analysis of multiple samples in a single step, without the requirement to collect output results from different sources and folders manually.

**Table 10:** Excerpt of the report table automatically generated by EAGER

Sample	# merged	% merged	# reads after C&M	# mapped reads	Duplicates
name	reads	reads	prior to mapping	prior to DeDup	removed with DeDup
3077	5,437,812	94.78 %	5,737,174	1,023,502	410,234
Jorgen625	12,956,100	80.5 %	16,093,580	2,659,178	94,005
Refshale16	32,041,091	73.61 %	43,528,407	12,782,665	3,872,555
SK2	51,364,343	99.27 %	51,741,310	48,211,553	47,353,377
SK8	7,683,942	72.5 %	10,597,941	1,227,067	185,913

The report shows results for five leprosy samples processed with EAGER. The number of merged reads, the percentage of merged reads as well as the number of duplicates removed can be seen for the respective samples. Note that this has been narrowed down to fit the page layout. A full report features more statistical values describing a sample, depending on which methods have been chosen to be executed in the pipeline (see Additional file 1)

### Software availability and requirements

The EAGER pipeline is available in several types of flavors. For testing, a VirtualBox-based image is available, with all the required tools that can be executed on any platform supporting VirtualBox [30]. Note that this has some performance drawbacks, so that this image should be used only for testing. For more advanced users, a manual is available from our website (it.informatik.uni-tuebingen.deit.informatik.uni-tuebingen.de) with instructions on how to set EAGER up on different kinds of Linux/Unix-based operating systems, such as CPU clusters where a Docker-based installation is not feasible, due to access rights for example. We were successfully able to run EAGER on systems with 4–8 GB of RAM and four CPUs, ranging up to workstations with 500 GB of RAM and 64 CPUs, as well as a typical cluster grid infrastructure. Many state-of-the-art methods are used less by end users because of several dependencies that need to be fulfilled before a provided software method can be used. Especially when dealing with newly designed workflows, end users are often faced with highly complex software packages that need to be installed, used, and maintained on their respective infrastructure of choice. Though most of EAGER has been developed in the Java programming language, which is portable to many different types of operating systems, there still exist several necessary tools in EAGER that need to be included in such an environment. Subsequently, an end user would be forced to install these tools by manually compiling them or finding and installing appropriate executable versions of these tools.

To overcome these dependency-related issues and hide most of the technical dependencies of the EAGER pipeline, a Docker-based image (docker.iodocker.io) with all the dependencies of EAGER has been set up. For end users, this means that there is a single requirement in the form of a working Docker installation necessary to run the EAGER pipeline, making the installation and setup as well as the maintenance of EAGER as easy as possible and less prone to error. A further improvement is the centralized architecture of the Docker-image-based system, as fixes for errors in the pipeline can be easily distributed to any installation worldwide. The users can then update their installation to any published revision of the pipeline with a single command at any time, while Docker guarantees that the image pulled from the server contains exactly the software the user wanted to pull. Furthermore, the EAGER images are stored in a tagged archive on our web server, enabling users to stay with older versions of the pipeline or step back to a previously published version of the pipeline at any given time point. This can be useful, for example, when results from former publications need to be reproduced. For some end users, the possibility of running Docker images on a cloud computing infrastructure, such as Amazon EC/2 or Google Cloud instances, might be a good alternative to buying and installing their own hardware, especially when the analysis of aDNA data is only done, e.g., on a per project basis and the computing resources would lie idle for most of the time. In such cases, the renting of an infrastructure as a service (IaaS) cloud computing unit together with the EAGER Docker image could be beneficial in terms of overall analysis costs. To enable administrators to install and set up the pipeline on different types of infrastructure, we also provide access to the executables used in the pipeline as well as the main pipeline components. These can be used to set up the pipeline, for example, on grid computing infrastructures that do not rely on Docker or cloud computing instances for task execution. Note that this requires end users to download and install all the subsequent tools used by the pipeline as well, making most of the installation more complex than the setup of solely a Docker container. A set of links to download the required tools for the EAGER pipeline as well as the Docker-based image of EAGER is available on our website (https://it.informatik.uni-tuebingen.deit.informatik.uni-tuebingen.de). EAGER and all its components are published under GPLv3, and the source code is available on GitHub (https://github.com/apeltzer/EAGER-GUI).

### Data availability

All ancient genome data sets are available from SRA and/or ENA (accession IDs in Table 1). To test our Clip&Merge tool, we used an artificial data set provided by the authors of FLASH, which can be downloaded from their webpage (https://ccb.jhu.edu/software/FLASH/). The modern data set to compare EAGER and PALEOMIX can be downloaded from bioplanet.com. Here, we used the illumina-100bp-pe-exome-30x data set available from GCAT. Finally, the simulated low coverage can be reproduced by merging BAM files from LBK1 to LBK8 into a single BAM file after mapping to hg19 and then sub-sampling from these BAM files with a random seed and varying *s*_*i*_=(0.005,0.01,0.02,0.05,0.10,0.20,0.30,0.40) using SAMtools (command ‘samtools view -s *s*_*i*_ input.bam > output.bam’.)

### Ethical statement

No ethical approval was required for this study.

## Additional file

Additional file 1 The following additional data are available with the online version of this paper. Additional file 1 contains the full report automatically produced by EAGER. (XLS 9.50 kb)
